# Estimating measurement equivalence of the 12-item General Health Questionnaire across ethnic groups in the UK

**DOI:** 10.1017/S0033291721003408

**Published:** 2023-04

**Authors:** Kirby King, Nick Allum, Paul Stoneman, Alexandru Cernat

**Affiliations:** 1 University of Essex, Colchester, UK; 2 Goldsmiths College, London, UK; 3 University of Manchester, Manchester, UK

**Keywords:** Ethnic minority groups, GHQ-12, measurement equivalence, scale reliability

## Abstract

**Background:**

This study investigates the extent to which the GHQ-12 exhibits configural, metric and scalar invariance across six ethnic groups in Britain and Northern Ireland, using the UK Household Longitudinal Study (*N* = 35 410).

**Methods:**

A confirmatory factor analysis was carried out on a white British group in order to establish an adequate measurement model. Secondly, a multi-group confirmatory factor analysis was conducted in order to assess measurement invariance. A sensitivity analysis comparing summated and latent means across groups was carried out. Finally, revised estimates of scale reliability were derived using two different methods.

**Results:**

A one-factor model including correlated error terms on the negatively phrased items showed superior fit in all ethnic groups. Tests for equal factor loadings and intercepts also showed adequate fit demonstrating metric and scalar invariance. Latent and summated scale estimates of mean group differences were similar for all groups. Scale reliability using McDonald's *ω* is lower than when using the more conventional Cronbach's *α*. Reliability across groups is reasonably consistent.

**Conclusions:**

We find that the GHQ-12 does not display obvious bias in regard to ethnic groups in the UK and that valid comparisons across these groups can be made for the purposes of population research. Caution is needed when using as a screening tool for individuals.

## Introduction

The General Health Questionnaire (GHQ) was developed in 1972 as a screening tool to identify those who are at risk of identifying psychiatric disorders (Romppel et al., 2017, p. 1). It was designed to identify deterioration in normal functioning and therefore focuses on common mental health problems such as anxiety, depression and social impairment, rather than severe illnesses such as schizophrenia or bipolar disorder (McDowell, 2006, p. 259). There are several versions of the GHQ in use, which can consist of 12, 28, 30 or 70 items (Jackson, 2007, p. 79). The GHQ-12 is one of the most widely used for both individual screening purposes and population research (Hankins, 2008). Concerns have been raised, however, about the relative lack of attention given to measurement equivalence *vis-a-vis* the GHQ-12 (French & Tait, 2004). When assessing the mental health profile of individuals and populations, it is assumed that the measurement properties of the survey items are consistent across different groups. That is to say, if differences in mental health are observed between different groups, these disparities are assumed to reflect real differences in health status not artefacts of measurement. This assumption, however, may not always hold. Latent factor structures may vary across groups; individual item loadings may differ across groups; and the estimated mean values of scales and subscales may also differ across social groups even though no real differences pertain. These issues of what are known in the literature as configural, metric and scalar invariance (Allum, Read, & Sturgis, 2018) are of practical importance for the following reason. As part of its long-term plan, the UK's National Health Service is committed to monitoring and improving mental health in all sections of society. A necessary implication is that for the GHQ-12 to play a role in this effort, it needs reliably to measure mental health profiles across, *inter alia*, different ethnic groups (NHS England, 2017, p. 14). Consequently, generating an instrument valid for all ethnic groups is an important step towards ensuring that such a target can be attained (Eisen, Gerena, Ranganathan, Esch, & Idiculla, 2006, p. 305).

The potential issue of measurement invariance across different ethnic groups is founded upon the ways in which differences in racial and ethnic identities affect how individuals report psychosocial functioning (Bowe, 2017, p. 90). The concern is that the measurement properties of health metrics, such as the GHQ-12, may differ across ethnic groups because each group defines the same health issue differently and uses different symptoms to identify it. If this is the case, a questionnaire developed to measure mental health for one group will fail to identify other aspects of the construct as understood by another group (Crockett, Randall, Shen, Russell, & Driscoll, 2005, p. 48). Furthermore, different ethnic groups may interpret the response options differently. For example, a score of 28 on a metric for one group may reflect a respondent being moderately distressed, whereas for another group this may reflect a respondent being severely distressed. A scoring system that is optimal for the first group may result in the under or overestimation of mental health for the second group and therefore comparisons of these scores could be misleading (Banh et al., 2012, p. 354). It is clearly important, then, for health metrics to be invariant to allow for comparisons across ethnic groups to be made. Statistics such as population means and regression coefficients can only be validly estimated if the measures on which they rely are found to be invariant across such different groups (Chen, 2008, p. 1005). The aim of this study is to assess the measurement invariance of the GHQ-12 with respect to adult members of six ethnic minority groups in the UK.

## Analysis plan

The following analyses focus on the GHQ-12 as it is currently the most popular version due to its brevity and ease of administration (Molina, Rodrigo, Losilla, & Vives, 2014, p. 1031; Romppel et al., 2017, p. 1). We use data from the UK to initially fit a measurement model for the 12 GHQ items for the majority white British group. Extensive literature supports one, two and three-factor models. This has important implications for measurement equivalence; using a suboptimal model will complicate the interpretation of the scores, which could lead to mistaken estimates of mental function (Smith, Fallowfield, Stark, Velikova, & Jenkins, 2010, p. 2). Evaluating the validity of the results from previous research, however, is complicated by the fact that results will be affected by the use of diverse samples and methods. In terms of the former, model solutions have been derived from data generated from both probability and non-probability samples, of varying sizes. In terms of the latter, researchers have taken different approaches to dealing with positive and negative items. While including both can deter acquiescence bias and provide for a better fitting multidimensional solution (Marsh, 1996, p. 810), Hankins (2008, p. 2), writing about the GHQ, questions whether the multi-factor solutions that have been derived in the literature have simply reflected the inclusion of positive and negative worded items rather any real multidimensionality of health status as measured by the instrument. In our analyses, we assess several alternative formulations that take account of such putative method effects in different ways.

Having fitted a measurement model which takes account of method effects in the white British group, we go on to perform a multi-group confirmatory factor analysis to test for measurement invariance using standard procedures. The only extant research that examines measurement invariance amongst ethnic groups for the GHQ-12 was concerned only with adolescents. Results from this work suggested measurement equivalence was a reasonable assumption but could not speak to adult populations (Banh et al., 2012; Bowe, 2017; Crockett et al., 2005). The present research, then, represents the first evaluation of the GHQ-12 as a viable multi-ethnic instrument for adults of all ages.

## Methods

The data for our analyses come from Wave 6 of Understanding Society, The UK Household Longitudinal Study (UKHLS). The survey employs a proportionately stratified, clustered probability sample design (McFall, Nandi, & Platt, 2016, p. 10). UKHLS includes an ethnic minority boost sample designed to yield around 1000 additional respondents from each of five minority groups: Indians, Pakistanis, Bangladeshis, Caribbeans and Africans, as well as a ‘mixed’ group The sample was restricted to those who completed the questionnaire in English (the overwhelming majority) so that cultural and language translation effects were not conflated (Prady et al., 2013, p. 12). This culminated in an analytical sample size of 35 437 of which 83% are white British (*n* = 29 432) and 17% are black and minority ethnic (BAME) (*n* = 6005), 2% identify as mixed race (*n* = 757), 4% are Indian (*n* = 1518), 4% are Pakistani (*n* = 1263), 2% are Bangladeshi (*n* = 567), 2% are Black Caribbean (*n* = 804) and 3% are Black African (*n* = 1096). Ethnic group membership is derived from asking respondents to say with which ethnic group they self-identify.

Individual GHQ items are sometimes recoded and analysed by collapsing the categories to form binary items (see, e.g. Padrón, Galán, Durbín, Gandarillas, & Rodríguez-Artalejo, 2012). Retaining the original four-point scale metric is the more common practice (Abubakar & Fischer, 2012; Bowe, 2017; Campbell & Knowles, 2007; Cheung, 2002; French & Tait, 2004; Graetz, 1991; Hankins, 2008; Hu, Stewart-Brown, Twigg, & Weich, 2007; Molina et al., 2014; Politi, Piccinelli, & Wilkinson, 1994; Romppel et al., 2017; Romppel, Braehler, Roth, & Glaesmer, 2013; Ye, 2009). We adopt the latter approach as there seems to be no advantage in discarding information by collapsing the items. The question wording and response scales for the measures of the GHQ used in the survey can be found in Appendix 1. We fit our models using maximum likelihood estimation in the Amos 25 software package (Arbuckle, 2017). Four-point ordinal items, as we have here, are suitable for this purpose (Bentler & Chou, 1987), although other estimation methods for ordered categorical variables are available that make different assumptions. As such, we also fitted our models using the weighted least-squares mean-variance adjusted (WLSMV) estimator in Mplus (Muthén & Muthén, 1998) as a sensitivity check. Our conclusions are robust to the choice of estimator, and we include the WLSMV estimates in online Supplementary material S1.

The first stage of our analysis is to estimate an appropriate baseline measurement model on the majority white British sample. We begin with the assumption that mental health as measured by GHQ-12 is substantively a unidimensional construct. Two alternative additions to this basic specification have been proposed in order to deal with response effects due to the mixture of positively and negatively worded items. These are the Correlated Trait, Correlated Uniqueness (CTCU) model and the Correlated Trait, Correlated Methods (CTCM) model (Lindwall et al., 2012). As applied to the GHQ-12, the CTCM model includes a specific latent method effect factor whereas the CTCU model introduces correlations amongst the error variances of the negatively worded items. The CTCM is more parsimonious but makes assumptions that may not hold in practice (Lance, Noble, & Scullen, 2002). Both models have been used in studies employing the GHQ-12. For example, Ye (2009) used the CTCM model whereas Hankins (2008) and Aguado et al. (2012) used the CTCU model. Molina et al. (2014) compared the fit of both models and found that they both fitted the data well, although the CTCU model fitted the data better.

**Fig. 1.**
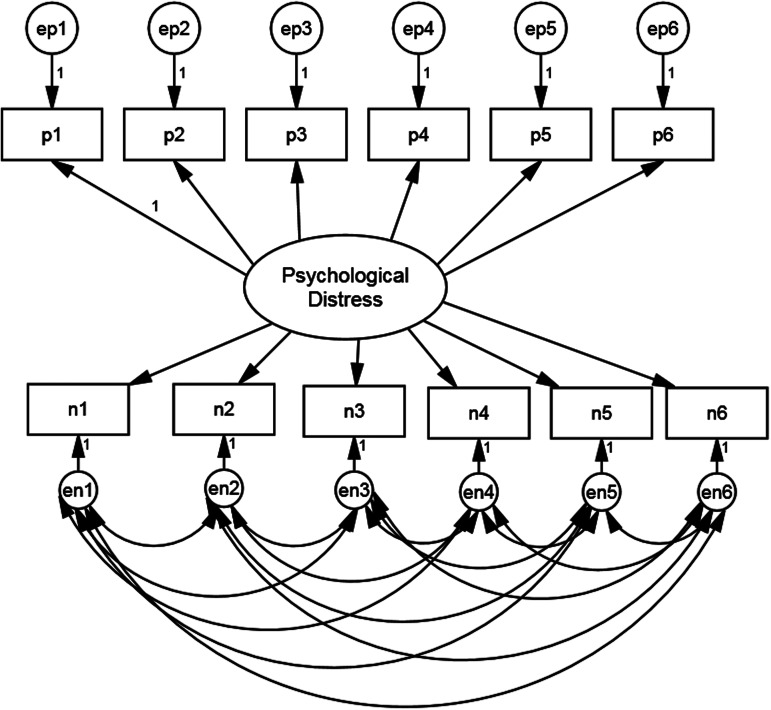
Model specification for GHQ-12 CTCU model.

**Fig. 2.**
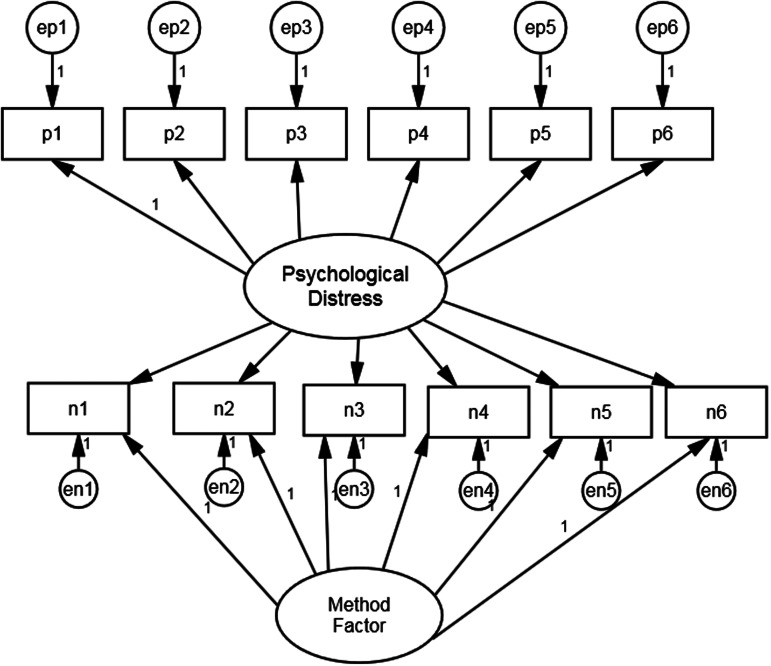
Model specification for GHQ-12 CTCM model.

We follow these authors and assess both CTCM and CTCU models. Figures 1 and 2 present the path diagrams for these models. Figure 1 shows the negatively phrased items with correlated error terms whereas Fig. 2 has an additional method factor, uncorrelated with the substantive factor. The loadings of the method factor are also constrained equal as there is no reason to believe that any one question is more vulnerable to a method effect than any other (Hankins, 2008).

After establishing a satisfactory baseline model, we go on to test for measurement invariance between the white British and BAME samples. We proceed by moving from less to more constrained models, assessing fit at each stage (Dimitrov, 2010, p. 125; Van der Velde & Saris, 2011, p.).

Specifically, we evaluate models with progressively more restrictive parameter constraints as follows. The configural invariance assesses whether the factor structure of the measurement model is the same across groups (Meuleman & Billiet, 2011, p. 186). At this most basic level, the covariances between GHQ-12 items must be reproducible with the same number of common factors across ethnic groups, and each common factor must be associated with identical item sets across group (Van de Velde, Levecque, & Bracke, 2009, p. 17). Next, we move to the metric invariance model, which tests the hypothesis that the factor loadings are equal across groups. The rationale for this restriction is as follows. In order to be able to compare the mental health of one group compared to another, it is essential that the meanings or interpretations of the questions are consistent between groups. Metric invariance describes the situation where the association between each item and the latent variable is the same for each group (Yap et al., 2014, p. 439). If this turns out to be the case, one can argue that the questions ‘hang together’ in the same way and that it furthermore makes sense to regard them as valid indicators of the same underlying mental health construct for members of each ethnic group.

Finally, we test for scalar invariance. This requires that not only the factor loadings but the intercepts of each item to be equivalent across ethnic groups (Davidov, Datler, Schmidt, & Schwartz, 2011, p. 150). That is to say, the expected score on an item for someone of, say, Indian ethnicity who is at the mean on the latent mental health variable (factor means are fixed at zero for identification purposes) should be the same as the expected score on that item for a member of the African group. If this condition is met for all items, it means that comparisons of latent mean levels across groups should be valid. Additionally, and perhaps equally importantly, if scalar invariance is demonstrated, the common practice of creating summated scale scores from the items should also lead to these measures being valid for making ethnic group comparisons of mean mental health levels.

## Results

Table 1 shows the goodness-of-fit measures for the two baseline measurement models. Both models included an accommodation for the presumed method effect associated with the negatively worded items. Unsurprisingly, with such a large sample size, neither model fits on the χ^2^ test statistic, as in both cases the critical value is exceeded. We instead rely on several measures of approximate fit as suggested by Hu and Bentler (1999), namely comparative fit index (CFI), Tucker–Lewis index (TLI), root mean square error of approximation (RMSEA) and standardised root mean residual (SRMR). On this basis, the CTCU model shows a good fit; CFI = 0.975; TLI = 0.958; RMSEA = 0.059; SRMR = 0.029. The CTCM model, on the other hand, indicates a much poorer fit, with only the SRMR fit statistic being acceptable (<0.08). These models are non-nested so we also compare the values of AIC and BIC which permit a direct comparison of the two models. This again indicates that the CTCU model fits better with smaller AIC and BIC values; AIC = 4166.573, BIC = 4489.849.

**Table 1.** Model fits of baseline model (white British) comparing CTCU and CTCM models

Model description	Chi2	df	RMSEA(90% CI)	CFI	TLI	SRMR	AIC	BIC
CTCU model	4088***	39	0.059(0.058–0.061)	0.975	0.958	0.029	4166	4489
CTCM model	9655***	53	0.078(0.077–0.080)	0.941	0.926	0.0379	9705	9912

† *p* < 0.10; * *p* < 0.05; ** *p* < 0.01; *** *p* < 0.001.

The CTCU baseline model was subsequently used as the basis for testing for invariance across ethnic groups.

Fit statistics are shown in Table 2. The configural invariance specification indicates a good fitting model. The fit indices from Table 2 demonstrate good fit at the configural level for all ethnic groups, CFI = 0.974; TLI = 0.955; RMSEA = 0.023; SRMR = 0.029.

**Table 2.** Model fits of free and constrained model and tests of measurement invariance

Model description	χ^2^	df	RMSEA (90% CI)	CFI	TLI	SRMR
Configural invariance	5586***	363	0.020 (0.020–0.021)	0.973	0.966	0.029
Metric invariance	5811***	429	0.019 (0.018–0.019)	0.972	0.970	0.029
Scalar invariance	6522***	501	0.018 (0.018–0.019)	0.969	0.971	0.029

† *p* < 0.10; * *p* < 0.05; ** *p* < 0.01; *** *p* < 0.001.

Table 3 shows the standardised factor loadings for the selected baseline model along with the intercepts. There is of course variation in the freely estimated loadings and intercepts but the aim of analysis is to determine whether the more constrained models provide plausible estimates of population parameters. Looking at Table 3, there is a reasonably consistent pattern of factor loadings across ethnic groups. Average standardised factor loadings for each group are in the low 60s with the rank ordering of items similar in each group. N1–N3 tend to load a little more weakly than the other items. This may be because they tap into specific behaviours rather than subjective states. Intercepts show similar patterns over each group except for the African group, which tends to show slightly lower estimated intercepts and the mixed group that shows higher intercepts for the negatively worded items. This group is heterogeneous and it is not possible to account for this observation in our models.

**Table 3.** Standardised factor loadings (*β*) and intercepts (*α*) for configural invariance model

	White British		Mixed		Indian		Pakistani		Bangladeshi		Caribbean		African	
	*β*	*α*	*β*	*α*	*β*	*α*	*β*	*α*	*β*	*α*	*β*	*α*	*β*	*α*
P1 – Able to concentrate	0.63	1.15	0.66	1.15	0.58	1.07	0.61	1.10	0.62	1.08	0.66	1.18	0.66	0.99
P2 – Playing a useful part in things	0.63	1.08	0.63	1.09	0.59	1.03	0.59	1.07	0.54	1.07	0.58	1.07	0.58	0.96
P3 – Capable of making decisions	0.64	1.03	0.67	1.02	0.57	0.98	0.63	0.98	0.63	0.97	0.62	0.99	0.62	0.88
P4 – Enjoy normal activities	0.70	1.14	0.74	1.16	0.73	1.10	0.76	1.13	0.73	1.12	0.71	1.17	0.71	1.03
P5 – Face up to problems	0.70	1.06	0.68	1.02	0.61	1.03	0.61	1.10	0.66	1.07	0.62	1.01	0.62	0.98
P6 – Reasonably happy	0.70	1.05	0.73	1.06	0.61	1.02	0.67	1.02	0.71	1.03	0.67	1.04	0.67	0.94
N1 – Lost much sleep over worry	0.51	0.80	0.55	1.00	0.53	0.87	0.60	0.93	0.50	0.93	0.61	0.97	0.61	0.85
N2 – Constantly under strain	0.53	0.96	0.59	1.15	0.56	0.97	0.60	0.98	0.59	0.96	0.60	1.05	0.60	0.94
N3 – Couldn't overcome difficulties	0.59	0.79	0.64	0.97	0.57	0.79	0.64	0.90	0.55	0.83	0.56	0.88	0.56	0.70
N4 – Unhappy or depressed	0.63	0.73	0.69	0.94	0.67	0.81	0.69	0.88	0.66	0.85	0.64	0.84	0.64	0.75
N5 – Losing confidence	0.62	0.68	0.66	0.80	0.63	0.65	0.68	0.73	0.64	0.67	0.63	0.71	0.63	0.55
N6 – Thinking of self as worthless	0.61	0.39	0.63	0.48	0.59	0.41	0.61	0.49	0.62	0.41	0.59	0.37	0.59	0.34

Consistent with these relatively small estimated group differences, the first constrained model, which tests for equal factor loadings, showed a good fit and little change at all from the congeneric model in the approximate fit indices. The difference in χ^2^ exceeds the critical value for statistical significance at the 5% level but in view of the large sample size, we do not consider this indicative of substantial loss of fit.

Next, the scalar invariance model was tested, by constraining both factor loadings and intercepts to equality across groups. These additional constraints again do not lead to substantial loss of fit, except on the χ^2^ difference between metric and scalar models, which again exceeds the critical value.

Given that the scalar invariance model turns out to be compatible with the data, this opens the way to being able to compare latent mean levels of mental health across ethnic groups.

To do this, we fitted an additional model, with the same specification as previously but where we fix the white British latent mean at zero and allow the other groups' means to be freely estimated in relation to the white British reference group. These estimates then represent the difference between white British average mental health and that for each other group. They are equivalent to the standardised effect size Glass's Delta, which is from the d-family of such statistics (Rosenthal & DiMatteo, 2001; Steinmetz, 2011). It is computed by dividing the difference between treatment and control group means (here the control group is the reference group, white British) by the standard deviation of the control group. For most practical situations where the GHQ-12 is put to use, a summated scale score is computed. We generated a new scale variable computed as the mean of each of the GHQ-12 items (with negatively worded items reverse coded) and then calculated Glass's delta for the differences between white British and each of the other groups on this new scale to generate a measure that is comparable to the latent difference. Both latent and observed standardised effect sizes are displayed in Table 4. The assumption of equal group variances is not required. The *p* values shown are derived from a *z*-test in the case of the latent means and a *t* test in the case of the summative score means.

**Table 4.** Ethnic group standardised differences in means for GHQ-12 (Glass's Δ)

	Standardised effect sizes (reference = white British)					
Glass's Δ	Mixed	Indian	Pakistani	Bangladeshi	Caribbean	African
Latent	0.08*	−0.09**	0.02	−0.04	0.02	−0.28***
Summative scale	0.18***	−0.02	0.09**	0.03	0.08*	−0.17***

† *p* < 0.10; * *p* < 0.05; ** *p* < 0.01; *** *p* < 0.001.

The size of the between-group differences varies somewhat between latent and summated scales. This is likely due in part to the fact that the latent estimates take account of the disparities in factor loadings between the items whereas the simple summated score gives equal weight to each item. None of the effect sizes is large and not all reach statistical significance. For the effects that are significant, both latent and summated score differences have the same sign. The only group for which the estimated differences diverge between latent and summated scale is the Bangladeshi group, and for both estimates, the magnitude is trivial and non-significant.

## Scale reliability

Typically, researchers use Cronbach's *α* to assess the reliability of psychological scales. There are no hard and fast rules for what an acceptable level of reliability is, although for applications where there are consequences for individuals, a higher standard of reliability is needed than where research is aiming only to capture group differences (Nunnally, 1978). However, *α* makes the assumption that each item has the same relationship to the underlying target construct (*τ*-equivalence, equal factor loadings) and that only one dimension is measured. Equation 1 gives the definition of *α*, where *k* is the number of items, *V*(*X_i_*) is the variance of item *i* and *V*(*O*) the variance of the observed sum.1
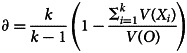
These assumptions are often not met, including in the present case, assuming our factor model is correct. An alternative measure of reliability, *ω*, was proposed by McDonald (1970), which takes into account varying factor loadings and is in fact a generalisation of *α*, as it does not require the assumption of *τ*-equivalence but reduces to *α* when this is assumed (Hayes & Coutts, 2020). Equation 2 defines *ω*, where *V*(*e_i_*) is the error variance in item *i* (from Equation 1); the summation is over all items. The factor loading for each item *i* is captured in *λ_i_*. Thus *ω* is the ratio of the sum of squared factor loadings to the sum of squared loadings and error variances.2
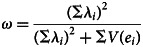
We computed *ω* for each ethnic group using the parameter estimates from the scalar invariance model using the Amos methods described in Hayes and Coutts (2020). We display these alongside the corresponding *α* in Table 5.

**Table 5.** Reliability estimates for GHQ-12 scale by ethnic group

	*α*	*ω*
White British	0.90	0.75
Mixed	0.91	0.81
Indian	0.89	0.76
Pakistani	0.91	0.79
Bangladeshi	0.90	0.75
Caribbean	0.90	0.79
African	0.87	0.68

It is readily apparent that *α* overestimates the reliability of the GHQ-12 in every ethnic group under the incorrect (in this case) assumption of *τ*-equivalence. The reason for this, as can be deduced from Equation 2, is that in the standard summated scale, the variance due to the substantive mental health construct is mixed with that due to the undifferentiated second dimension, acquiescence response bias.

Our model partials this out through the specification of correlated errors on the negatively worded items. This means that the ratio of substantive to total variance is (correctly) attenuated using *ω* to estimate reliability. Consistent with the rest of our analysis, there is little variation in *ω* across groups except for the African respondents, for whom reliability is somewhat lower on both measures.

## Discussion

The results of this study demonstrate support for previous studies that have estimated a unidimensional structure of the GHQ-12 once response bias on the negatively worded items is taken into account (Aguado et al., 2012; Hankins, 2008; Molina et al., 2014; Rey, Abad, Barrada, Garrido, & Ponsoda, 2014; Romppel et al., 2013). Further, the results indicate that the multidimensional models such as the three-factor model proposed by Graetz is the likely result of the inclusion of the positive and negative worded items in the questionnaire. We see no reason to consider the GHQ-12 as capturing more than one dimension of mental health, which partly supports the practice in psychological, clinical and epidemiological research of using a standard summated scale approach.

That said, we do find non-trivial response effects due to the inclusion of negative and positive items and we fit models to correct for this. In this regard, our results replicate the findings of previous studies that suggest that the CTCU model is appropriate (Aguado et al., 2012; Hankins, 2008) rather than alternative recommendations (e.g. Lance et al., 2002) to use the more parsimonious CTCM model. Our data come from a data-generated large, representative random probability sample of a heterogeneous population and it appears that the less restrictive CTCM model is better able to capture method effects that may be multidimensional – to include, for example, question order effects (Lindwall et al., 2012, p. 201; Molina et al., 2014, p. 1035).

In line with research on adolescents, our results suggest that using the GHQ-12 in the UK across different ethnic adult groups is unlikely to lead to grossly unreliable conclusions. Comparisons between these groups also appear to be justified. These results are consistent with other studies that have examined measurement equivalence with respect to ethnic groups of other mental health instruments (Banh et al., 2012; Crockett et al., 2005; Eisen et al., 2006; Kim, Sellbom, & Ford, 2014). An exception is Prady et al. (2013) who failed to find invariance using the GHQ-28. However, here the population was pregnant women in a clinical setting and several languages were also used to translate items. In our data, a broader population answered questions as part of a general survey – a very different context. Our conclusions may not hold when using the GHQ-12 in a clinical setting if the items are interpreted in very different ways to the way people answer questions as part of a survey interview in the home. More research comparing these contexts of administration would be useful, both quantitative and qualitative.

A sensitivity analysis where we compared mean differences between groups on both latent and summated scale estimators indicated that similar conclusions would be reached using either formulation, with the pattern of statistical significance and direction being isomorphic. The use of structural equation modelling with latent means will in general permit smaller true differences across groups to be detected, but we do not believe that health researchers will be seriously misled by using a summated scale.

On a less encouraging note, we observed quite substantial differences in estimates of the reliability of the GHQ-12 comparing two different measures, Cronbach's *α* and McDonald's *ω*. However, similar magnitudes of difference are evident in all ethnic groups. We conclude that the conventional use of *α* to estimate the scale reliability of the GHQ-12 may lead to more optimistic assessments than are justifiable, and that caution should be exercised particularly where the instrument is to be used as a screening tool for individuals. More research could examine the predictive validity of the recommended caseness threshold of 3 in the 12-item version of the GHQ in light of this alternative reliability measure, and whether this threshold is appropriate for all ethnic groups.

## Conclusion

The purpose of this study has been to directly inform researchers and policy makers on whether we can reliably and accurately estimate the mental health profiles of different ethnic groups using the GHQ-12, a commonly-used instrument. Our results are broadly in the affirmative. An important advantage of our approach compared to previous research stems from the use of data from an adult population, generated from a large representative probability sample. This contrasts with the use of non-probability or special population samples in previous work in this area. Our results indicate that, for the adult UK population, the GHQ-12 can be used to assess mental health within and between a range of ethnic groups. We caveat this by pointing to the lower than previously assumed reliability of the scale and for this reason it may be prudent to consider longer versions of the scale for use as a screening tool for individuals.

## Supplementary material

For supplementary material accompanying this paper visit https://doi.org/10.1017/S0033291721003408. click here to view supplementary material

## Appendix 1

Table A1 presents the measures of the GHQ used in the survey, with a higher score indicating the most distressed. In order to ease interpretation, the six negatively-phrased items were labelled n1 to n6, and the six positively phrased items were labelled p1 to p6.

**Table A1.** Item wordings, response scales, and variable names

Item wording and response scale
**p1:** Have you recently been able to concentrate on whatever you're doing? (0 = Better than usual, 3 = Much less than usual)
**n1:** Have you recently lost much sleep over worry? (0 = Not at all, 3 = Much more than usual)
**p2:** Have you recently felt that you were playing a useful part in things? (0 = More so than usual, 3 = Much less than usual)
**p3:** Have you recently felt capable of making decisions about things? (0 = More so than usual, 3 = Much less capable)
**n2:** Have you recently felt constantly under strain? (0 = Not at all, 3 = Much more than usual)
**n3:** Have you recently felt that you couldn't overcome your difficulties? (0 = Not at all, 3 = Much more than usual)
Item wording and response scale
**p4:** Have you recently been able to enjoy your normal day-to-day activities? (0 = More so than usual, 3 = Much less than usual)
**p5:** Have you recently been able to face up to problems? (0 = More so than usual, 3 = Much less able)
**n4:** Have you recently been feeling unhappy or depressed? (0 = Not at all, 3 = Much more than usual)
**n5:** Have you recently been losing confidence in yourself? (0 = Not at all, 3 = Much more than usual)
**n6:** Have you recently been thinking of yourself as a worthless person (0 = Not at all, 3 = Much more than usual)
**p6:** Have you recently been feeling reasonable happy, all things considered? (0 = More so than usual, 3 = Much less than usual)
