# Supplementary material for: Estimating measurement equivalence of the 12-item General Health Questionnaire across ethnic groups in the UK
Source: Psychol Med. 2021 Sep 9;53(5):1778–86. doi: 10.1017/S0033291721003408 (PMC10106293; doi:10.1017/S0033291721003408)
Supplement: Supplementary file 1 [file S0033291721003408sup001.pdf]

Supplementary material S1

Categorical equivalence with GHQ by ethnicity

To estimate the models we have made the following restrictions: - first loading fixed to 1 in all groups - thresholds of first variable equal across groups - first threshold of each of the other variables equal across groups - means were fixed as 0 for all models and variance to 1 in the reference group (white)

This is based on advice from: Millsap and Yun-Tein (2004), Millsap (2012).

The diff test is based on the Mplus one that takse into account the WLSMV estimation.

The models include correlations between negative worded items that are constrained to be equal across groups. All coefficients are unstandardised.

Model comparison

Scale	Model	Chi2	df	CFI	RMSEA	Chi2_diff	Chi2_diff_df	Chi2_dfff_p
ghq	configural	9613.680	436	0.982	0.065	NA	NA	NA
ghq	metric	7437.323	502	0.987	0.052	427.146	66	0
ghq	scalar	12941.078	647	0.976	0.061	5967.664	145	0

Coefficients from configural model

	AFRICAN	BANGLADESHI	CARIBBEAN	INDIAN	MIXED	PAKISTANI	WHITE
concentration	1.00	1.00	1.00	1.00	1.00	1.00	1.00
lossofsleep	0.78	0.58	0.72	0.77	0.63	0.88	0.66
usefulrole	1.12	0.66	0.73	0.98	0.86	0.93	0.96
makingdecisions	1.20	0.81	0.84	0.91	0.98	0.98	1.05
understrain	0.84	0.85	0.74	0.93	0.70	0.96	0.71
overcomingdifficulties	0.76	0.66	0.61	0.89	0.80	0.96	0.83
enjoyactivities	1.73	1.36	1.14	1.55	1.28	1.65	1.27
faceproblems	1.35	0.89	0.84	0.96	0.97	0.94	1.18
unhappy	0.97	0.97	0.80	1.27	1.00	1.18	0.97
losingconfidence	1.00	0.93	0.81	1.10	0.94	1.17	0.91
believeworthless	0.87	0.88	0.81	1.02	0.93	0.98	0.93
generalhappiness	1.32	1.11	1.01	1.14	1.26	1.21	1.28

How about the thresholds. Some are equal to make sure the model estimates (see above).

param	AFRICAN	BANGLADESHI	CARIBBEAN	INDIAN	MIXED	PAKISTANI	WHITE
concentration$1	-1.63	-1.63	-1.63	-1.63	-1.63	-1.63	-2.62
concentration$2	1.30	1.30	1.30	1.36	1.30	1.25	1.42
concentration$3	2.65	2.65	2.65	2.67	2.65	2.44	2.83
lossofsleep$1	-0.42	-0.42	-0.42	-0.42	-0.42	-0.42	-0.31
lossofsleep$2	0.98	0.93	0.96	1.09	0.83	0.96	1.18
lossofsleep$3	1.79	1.72	1.71	1.99	1.84	1.89	2.19
usefulrole$1	-1.46	-1.46	-1.46	-1.46	-1.46	-1.46	-1.92
usefulrole$2	1.56	1.35	1.25	1.54	1.29	1.37	1.57
usefulrole$3	2.59	2.00	2.16	2.69	2.29	2.28	2.62
makingdecisions$1	-1.37	-1.37	-1.37	-1.37	-1.37	-1.37	-2.19
makingdecisions$2	1.88	1.73	1.65	1.80	1.66	1.66	1.99
makingdecisions$3	1.18	1.31	1.16	1.31	0.95	1.30	3.24
understrain$1	-0.67	-0.67	-0.67	-0.67	-0.67	-0.67	-0.68
understrain$2	1.18	1.31	1.16	1.31	0.95	1.30	1.00
understrain$3	1.93	2.19	1.89	2.19	1.80	2.01	2.17
overcomingdifficulties$1	-0.38	-0.38	-0.38	-0.38	-0.38	-0.38	-0.25
overcomingdifficulties$2	1.19	1.15	1.13	1.30	1.07	1.16	1.49
overcomingdifficulties$3	2.00	2.02	1.95	2.37	2.21	2.16	2.56
enjoyactivities$1	-2.10	-2.10	-2.10	-2.10	-2.10	-2.10	-2.69
enjoyactivities$2	1.79	1.52	1.27	1.63	1.37	1.56	1.61
enjoyactivities$3	2.97	3.05	2.79	3.18	3.03	3.10	3.09
faceproblems$1	-1.57	-1.57	-1.57	-1.57	-1.57	-1.57	-2.48
faceproblems$2	1.70	1.44	1.58	1.57	1.72	1.28	2.04
faceproblems$3	2.76	2.26	2.68	2.65	2.93	2.21	3.32
unhappy$1	-0.30	-0.30	-0.30	-0.30	-0.30	-0.30	-0.27
unhappy$2	1.27	1.20	1.09	1.40	1.04	1.15	1.30
unhappy$3	2.19	2.23	2.08	2.58	2.20	2.21	2.54
losingconfidence$1	0.06	0.06	0.06	0.06	0.06	0.06	-0.03
losingconfidence$2	1.53	1.41	1.36	1.56	1.16	1.36	1.43
losingconfidence$3	2.39	2.37	2.32	2.53	2.40	2.47	2.53
believeworthless$1	0.70	0.70	0.70	0.70	0.70	0.70	0.76
believeworthless$2	1.82	1.85	1.83	1.91	1.63	1.66	1.95
believeworthless$3	2.38	2.42	2.61	2.57	2.68	2.49	2.83
generalhappiness$1	-1.47	-1.47	-1.47	-1.47	-1.47	-1.47	-2.05
generalhappiness$2	1.67	1.59	1.52	1.59	1.57	1.56	1.84
generalhappiness$3	2.82	2.74	2.84	2.69	3.22	2.74	3.18

We can also transform the thresholds in probabilities. The way to interpret it is the proportion of people in the previous category based on the model. For the first variable you have 5% in the first category, 85% in the second, 10% in the third and close to 0% in the last.

param	AFRICAN	BANGLADESHI	CARIBBEAN	INDIAN	MIXED	PAKISTANI	WHITE
concentration$1	0.05	0.05	0.05	0.05	0.05	0.05	0.00
concentration$2	0.90	0.90	0.90	0.91	0.90	0.89	0.92
concentration$3	1.00	1.00	1.00	1.00	1.00	0.99	1.00
lossofsleep$1	0.34	0.34	0.34	0.34	0.34	0.34	0.38
lossofsleep$2	0.84	0.82	0.83	0.86	0.80	0.83	0.88
lossofsleep$3	0.96	0.96	0.96	0.98	0.97	0.97	0.99
usefulrole$1	0.07	0.07	0.07	0.07	0.07	0.07	0.03
usefulrole$2	0.94	0.91	0.89	0.94	0.90	0.91	0.94
usefulrole$3	1.00	0.98	0.98	1.00	0.99	0.99	1.00
makingdecisions$1	0.09	0.09	0.09	0.09	0.09	0.09	0.01
makingdecisions$2	0.97	0.96	0.95	0.96	0.95	0.95	0.98
makingdecisions$3	0.88	0.90	0.88	0.90	0.83	0.90	1.00
understrain$1	0.25	0.25	0.25	0.25	0.25	0.25	0.25
understrain$2	0.88	0.90	0.88	0.90	0.83	0.90	0.84
understrain$3	0.97	0.99	0.97	0.99	0.96	0.98	0.98
overcomingdifficulties$1	0.35	0.35	0.35	0.35	0.35	0.35	0.40
overcomingdifficulties$2	0.88	0.87	0.87	0.90	0.86	0.88	0.93
overcomingdifficulties$3	0.98	0.98	0.97	0.99	0.99	0.98	0.99
enjoyactivities$1	0.02	0.02	0.02	0.02	0.02	0.02	0.00
enjoyactivities$2	0.96	0.94	0.90	0.95	0.91	0.94	0.95
enjoyactivities$3	1.00	1.00	1.00	1.00	1.00	1.00	1.00
faceproblems$1	0.06	0.06	0.06	0.06	0.06	0.06	0.01
faceproblems$2	0.96	0.93	0.94	0.94	0.96	0.90	0.98
faceproblems$3	1.00	0.99	1.00	1.00	1.00	0.99	1.00
unhappy$1	0.38	0.38	0.38	0.38	0.38	0.38	0.39
unhappy$2	0.90	0.88	0.86	0.92	0.85	0.87	0.90
unhappy$3	0.99	0.99	0.98	1.00	0.99	0.99	0.99
losingconfidence$1	0.52	0.52	0.52	0.52	0.52	0.52	0.49
losingconfidence$2	0.94	0.92	0.91	0.94	0.88	0.91	0.92
losingconfidence$3	0.99	0.99	0.99	0.99	0.99	0.99	0.99
believeworthless$1	0.76	0.76	0.76	0.76	0.76	0.76	0.78
believeworthless$2	0.97	0.97	0.97	0.97	0.95	0.95	0.97
believeworthless$3	0.99	0.99	1.00	0.99	1.00	0.99	1.00
generalhappiness$1	0.07	0.07	0.07	0.07	0.07	0.07	0.02
generalhappiness$2	0.95	0.94	0.94	0.94	0.94	0.94	0.97
generalhappiness$3	1.00	1.00	1.00	1.00	1.00	1.00	1.00
